# Effect of ocean acidification on the growth, response and hydrocarbon degradation of coccolithophore-bacterial communities exposed to crude oil

**DOI:** 10.1038/s41598-023-31784-5

**Published:** 2023-03-27

**Authors:** Afiq Mohd Fahmi, Stephen Summers, Martin Jones, Bernard Bowler, Sebastian Hennige, Tony Gutierrez

**Affiliations:** 1 School of Engineering and Physical Science, Heriot-Watt University, Edinburgh, EH14 4AS UK; 2 Fakulti Sains dan Sekitaran Marin, Universiti Malaysia Terengganu, 21030 Kuala, Terengganu Malaysia; 3 The Singapore Centre for Environmental Life Sciences Engineering and the School of Biological Sciences, Nanyang Technological University, Singapore, 637551 Singapore; 4 School of Civil Engineering and Geosciences, Newcastle University, Newcastle Upon Tyne, NE17RU UK; 5 School of Geosciences, University of Edinburgh, Edinburgh, EH9 3JW UK

**Keywords:** Microbial communities, Environmental microbiology, Climate sciences

## Abstract

Hydrocarbon-degrading bacteria, which can be found living with eukaryotic phytoplankton, play a pivotal role in the fate of oil spillage to the marine environment. Considering the susceptibility of calcium carbonate-bearing phytoplankton under future ocean acidification conditions and their oil-degrading communities to oil exposure under such conditions, we investigated the response of non-axenic *E. huxleyi* to crude oil under ambient versus elevated CO_2_ concentrations. Under elevated CO_2_ conditions, exposure to crude oil resulted in the immediate decline of *E. huxleyi*, with concomitant shifts in the relative abundance of *Alphaproteobacteria* and *Gammaproteobacteria*. Survival of *E. huxleyi* under ambient conditions following oil enrichment was likely facilitated by enrichment of oil-degraders *Methylobacterium* and *Sphingomonas,* while the increase in relative abundance of *Marinobacter* and unclassified *Gammaproteobacteria* may have increased competitive pressure with *E. huxleyi* for micronutrient acquisition*.* Biodegradation of the oil was not affected by elevated CO_2_ despite a shift in relative abundance of known and putative hydrocarbon degraders. While ocean acidification does not appear to affect microbial degradation of crude oil, elevated mortality responses of *E. huxleyi* and shifts in the bacterial community illustrates the complexity of microalgal-bacterial interactions and highlights the need to factor these into future ecosystem recovery projections.

## Introduction

Marine eukaryotic phytoplankton (microalgae) contribute significantly to some key global processes, including approximately half of global carbon fixation^1^ and approximately half of the oxygen in the atmosphere^2^. As key members at the base of the food chain, they play a fundamental role in the ecology of marine ecosystems. Together with their bacterial symbionts, which are found associated with their microalgal hosts at the cell surface (i.e. the phycosphere), these algal–bacterial communities are a major source and recycler of organic and inorganic carbon and nutrients^3–5^. Bacteria associated with microalgae have been posited to utilise algal exudates as carbon and energy sources^6,7^, whereas the algal hosts can benefit from bacterial-mediated trace metal/nutrient bioavailability^8^. A mutual sharing of iron and fixed carbon was shown to occur between several species of microalgae and bacteria^9^. Another study demonstrated that the supply of bacterial-produced vitamin B12 to the microalgal host occurred in exchange for fixed carbon^10^. It is also noteworthy that very few microalgal species can be maintained or sub-cultured in the laboratory for long periods in the absence of their bacterial symbionts (i.e. in an axenic state), testament to the pivotal role that the associated bacterial community plays in their overall success. However, studies aimed at assessing the impact of future ocean conditions on microalgae have largely employed the use of axenic species, or non-axenic individual species or consortia where the focus was on either the microalgae or the bacteria, rather than both as a collective holobiont (e.g. Joint et al.^11^).

The microbiology of the ocean, likely at all levels of habitation, are and will continue to be subject to anthropogenic-induced global change, with temperature and CO_2_ increases^12,13^. Of particular interest, the impact of future ocean conditions on coccolithophores has been extensively studied in recent years due to the potential sensitivity of these organisms to ocean acidification (OA)^14,15^. Despite OA affecting calcification of coccoliths, field studies have demonstrated an increase in abundance of coccolithophores in the subtropical region in recent years due to CO_2_ enrichment^16,17^. Laboratory studies of the coccolithophore *E. huxleyi* reported inconsistencies in response to OA due to strain specific responses as a result of pan-genomic variability^16,18,19^, which likely accounts for their global distribution and abundance during phytoplankton blooms.

*E. huxleyi* produces organic carbon compounds such as alkenone lipids, long-chained alkenes, long-chained alkenones, phospholipids, and glycolipids that all bare similarities to hydrocarbons that are constituents of crude oil^20,21^. As such, hydrocarbon-degrading bacteria have commonly been found living associated with *E. huxleyi*^9,22^, as well as with other species of eukaryotic phytoplankton^23–27^. Coccolithophores such as *E. huxleyi*, have also been found to harbour a bacterial community that is more diverse than that of dinoflagellates and diatoms, possibly due to the biochemically and biophysically more complex environment associated with the cell surface of actively growing and calcifying coccolithophores^22^. As *E. huxleyi* thrives in oligotrophic expanses of the open ocean, they could be a significant seed source of hydrocarbon-degrading bacteria in low-nutrient surface waters, as well as from the subsurface to the seafloor if these bacteria remain attached to dead coccolith cells and survive vertical sedimentation to the seafloor as marine snow.

The total quantity of underground oil reserves around the world is finite, so the rate of its production, which also accounts for the rate of discovering new underground oil reserves, has been in a steady state of decline for decades now. Consequently, the oil and gas industry has intensified its interests to exploring for oil in more challenging environments that include the Arctic, the subarctic, and in ever deeper waters. All these environments carry risk of disaster and the Deepwater Horizon oil spill is testament to this, which resulted in approximately 700,000 tonnes (4.9 million barrels) of Louisiana light sweet crude oil discharged into the Gulf of Mexico from a blown-out wellhead at a depth of ~ 1500 m^28,29^. Crude oil is a heterogenous mixture of thousands of chemical compounds grouped as saturates, aromatics, resins, and asphaltenes that can cause acute or chronic toxicity to a wide variety of sea life. Crude oil toxicity to microalgae can include impairment of cell permeability, loss of cell nuclei and cell mobility, CO_2_ absorption capacity, alterations to protein content, shrinkage of chloroplasts and pyrenoids, disruption of nuclei acid synthesis, and damage to DNA and RNA from oxidative stress^30–35^.

In the absence of national and international government intervention to prevent the oil and gas industry from exploring for oil in deeper waters and polar/subpolar environments, the likelihood of oil spills occurring in oligotrophic environments will inevitably be much higher than it has ever been. It is therefore imperative to gain foresight on how microbial communities might respond to crude oil spillage under future OA conditions. In this study, we investigated the growth and physicochemical changes in *E. huxleyi*, as well as shifts to its associated bacterial community to crude oil under future OA conditions. Our results provide new understanding on the future risk of crude oil spillage in the open ocean, where the synergistic or additive responses of these communities will be an underpinning factor in the resilience of the ocean to recovering from oil pollution in a future climate.

## Results

### ***E. huxleyi*** response to oil enrichment in elevated CO_2_ conditions

**Figure 1:**
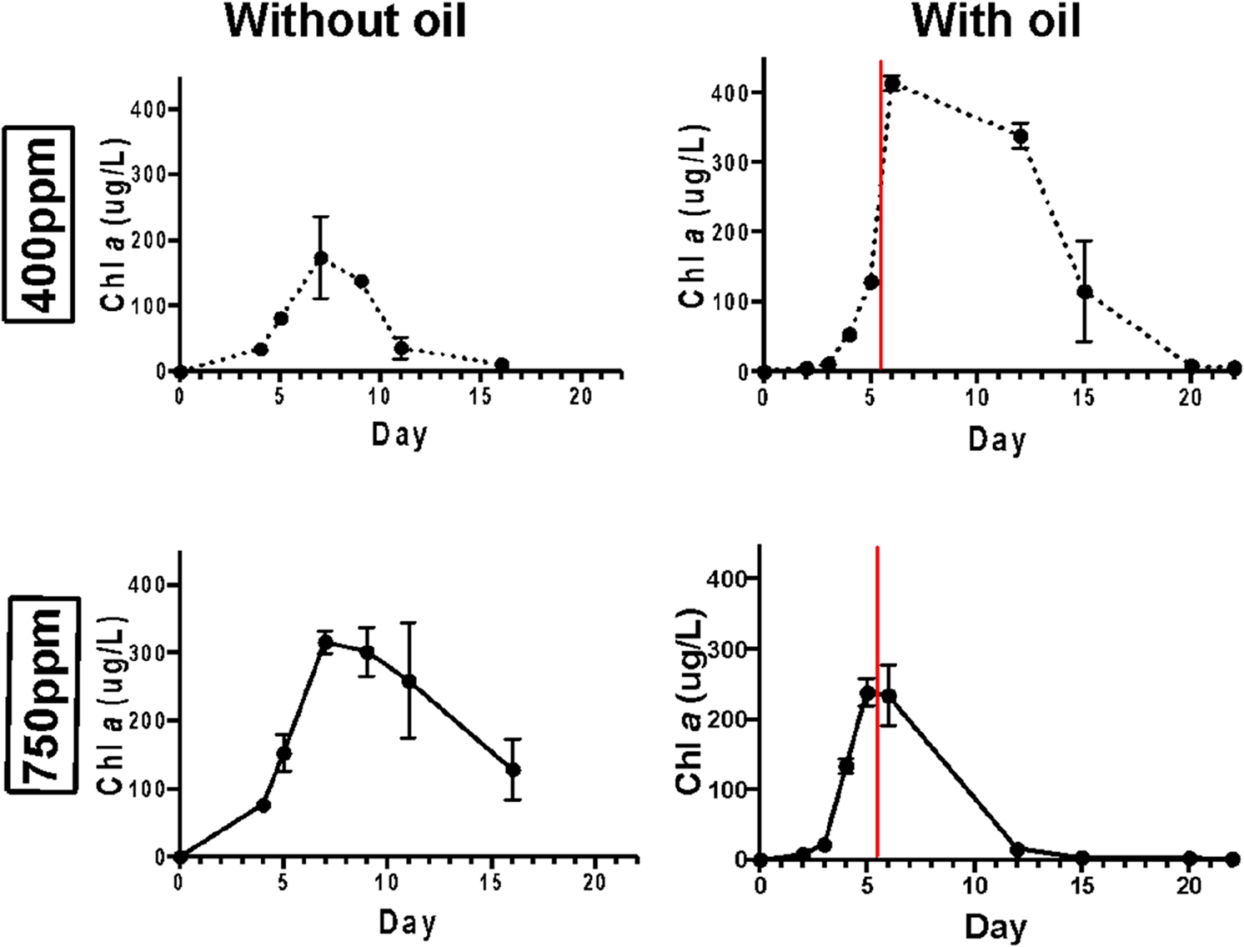
Growth response of *E. huxleyi*, measured by monitoring Chl *a* concentrations, in microcosms treated with ambient (400 ppm) and elevated (750 ppm) CO_2_. Red line indicates the timepoint (day 5) at which crude oil was added to the oil-amended microcosms. Values are averages of triplicate incubations ± standard error.

Changes in Chl *a* were used to determine the dynamics of *E. huxleyi* in response to crude oil under ambient and elevated CO_2_ conditions (Fig. 1). Distinct differences in the growth dynamic of the alga occurred between these conditions. Initially, Chl *a* concentrations (proxy for *E. huxleyi* growth) in elevated CO_2_ (750 ppm) microcosms were not significantly different to those in ambient CO_2_-treated microcosms on day 2 or 3 (*p* = 0.994, and *p* = 0.9644). On days 4 and 5, Chl *a* concentrations were significantly higher in elevated CO_2_-treated microcosms (*p* = 0.0002, *p* < 0.0001). In oil-amended microcosms, Chl *a* concentrations under ambient CO_2_ conditions continued to increase exponentially for an additional 24 h after oil enrichment at day 5, whereas in the elevated CO_2_ microcosms Chl *a* concentrations ceased increasing following oil enrichment. Chl *a* concentrations in the ambient microcosms reached significantly higher levels at days 12 and 15 (*p* < 0.0001 and *p* < 0.0001), and remained as such until the termination of these experiments. In contrast, Chl *a* concentrations after oil enrichment in the elevated CO_2_ microcosms decreased to below detection levels after day 12.

### Bacterial community dynamics in response to crude oil under ambient and elevated CO_2_ conditions

**Figure 2:**
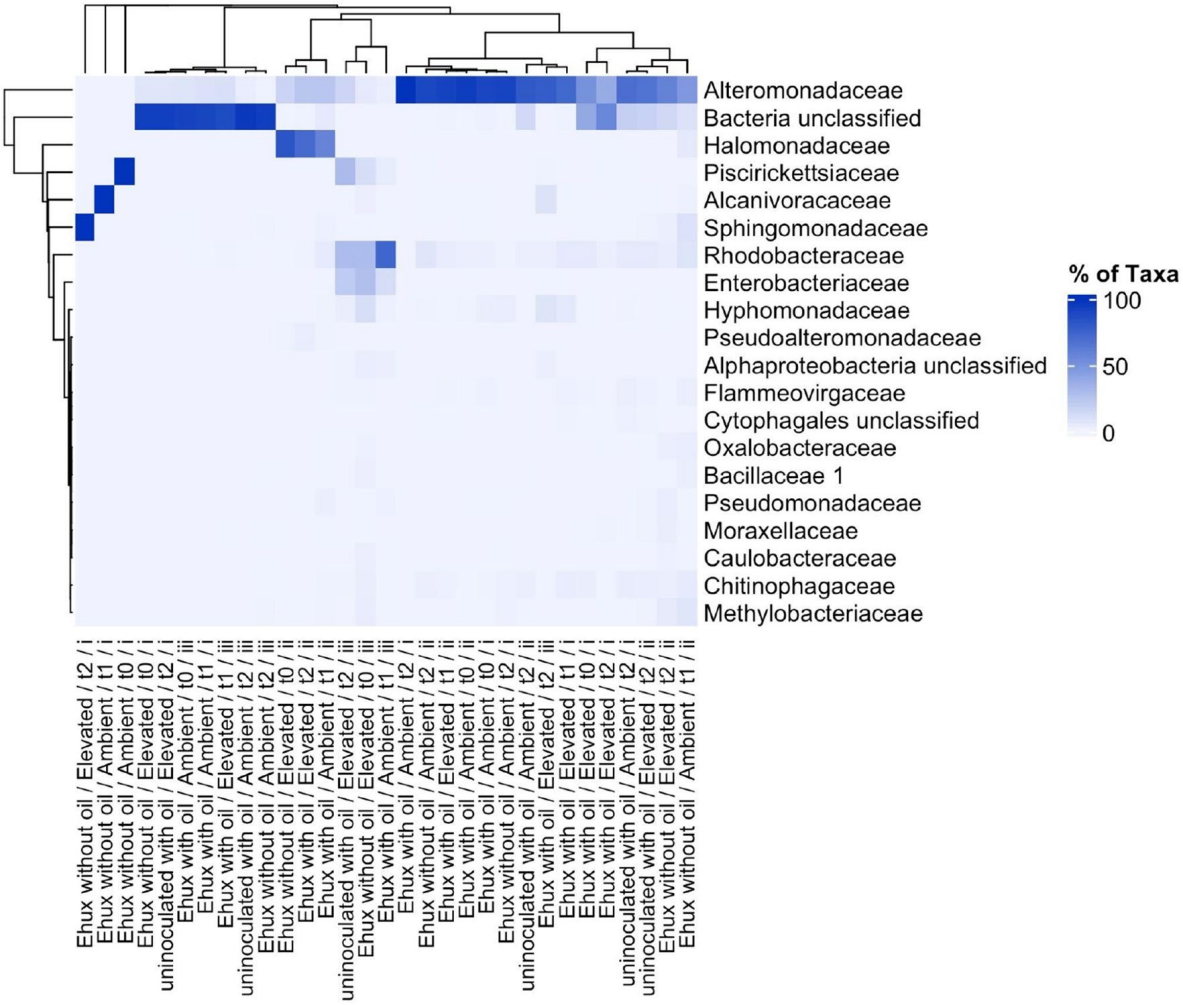
Heatmap of the bacterial OTUs that became enriched in incubations of *E. huxleyi* treated to ambient or elevated CO_2_ concentrations with or without exposure to crude oil at timepoints t_0_, t_1_ and t_3_ (described in main text). Uninoculated treatments represent those that were not inoculated with *E. huxleyi* (uninoculated controls). OTUs were considered enriched if there was a mean increase of at least 1% relative abundance (e.g., shift from 1 to 2%) in at least one time point for replicate incubations (i, ii, iii), and if the difference was statistically significant (*p* < 0.05). Colour key indicates square-root normalized relative abundance (%).

MiSeq sequencing of all samples (including triplicates) returned a total of 4,463,167 individual sequence reads. After pre-processing (merging, trimming, short-read and quality filtering), demultiplexing, denoising/clustering, chimera filtration and applying an OTU clustering cut-off of 97% for sequence similarity, 1,138 good-quality OTUs were obtained. This included 7 putatively novel taxa belonging to the classes *Gammaproteobacteria* and *Alphaproteobacteria*, and the families *Rhodobacteraceae*, *Halomonadacea*e, *Alteromonadaceae* and *Veillonellaceae*, and to an unclassified bacterial taxon. Species richness of the bacterial communities across treatments showed similar evenness in Shannon diversity index (*p* > 0.05; Supplementary Fig. 1). A non-metric multidimensional scaling (nMDS) ordination plot showed at least 60% similarity of the communities across all treatments (Supplementary Fig. 2). A heatmap showing the bacterial community at family level (Fig. 2) shows similarity of taxa distribution were not clustered depending on CO_2_ levels or oil exposure. ANOSIM pairwise test identified no statistical differences (*p* > 0.05) in the beta diversity of the bacterial communities, based on the Bray–Curtis dissimilarity matrix, between all treatments, thus supporting the results in Supplementary Fig. 2 by nMDS indicating that OA conditions (with reference to the elevated CO_2_ treatments) and exposure to crude oil does not significantly affect the diversity and composition of the bacterial community associated with *E. huxleyi*.

**Figure 3:**
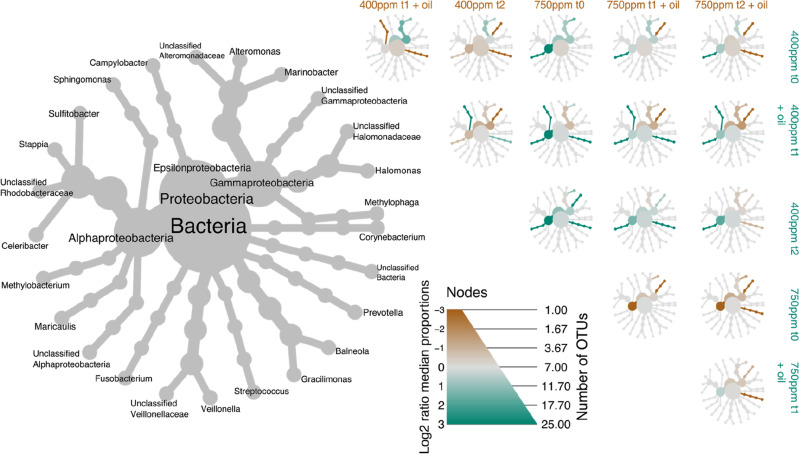
Heat-tree plot of bacterial community associated with *E. huxleyi* in different CO_2_ and oil enrichment treatments. Larger grey tree represents taxon labelled key of the smaller heat trees showing pairwise comparison between treatments. Diameter of nodes shows qualitative number of OTU of each taxon. Colour intensity of smaller trees (green or brown) corresponds to significantly abundant taxons between treatments determined with Wilcox rank-sum test followed by Benjamini Hochberg (FDR) correction for multiple comparisons. Treatments correspond to CO_2_ acclimation at 400 ppm (ambient) or 750 ppm (elevated) at different timepoints. Timepoints: t_0_ represents day 5 since the start of the incubations and just prior to oil enrichment, t_1_ represent day 12 since the start of the incubations or 7 days after oil enrichment, and t_2_ represents day 19 since the start of the incubations or 14 days after oil enrichment.

Further analysis by pairwise comparison (log_2_ median ratio) of bacterial taxa between treatments displayed as heat trees (Fig. 3) showed significant shifts in relative abundance of taxa within the community between treatments. Under ambient CO_2_ conditions, the bacterial community associated with *E. huxleyi* were dominated by members belonging to the genus *Marinobacter* (29% relative abundance), followed by unclassified members of the class *Gammaproteobacteria* (11%), the genus *Methylobacterium* (8%), the class *Alphaproteobacteria* (6%) and to unclassified bacteria (6%) (Supplementary Table 1). Prior to oil enrichment, *Marinobacter* and *Methylobacterium* were significantly higher in relative abundance in ambient CO_2_ microcosms compared to in microcosms with elevated CO_2_, while no taxa were found enriched under elevated CO_2_ conditions compared to ambient treated microcosms prior to oil enrichment (Fig. 3). In comparison, the bacterial community in the elevated CO_2_ treatments was dominated by *Marinobacter* (15%), *Methylobacterium* (11%), *Sphingomonas* (8%) and unclassified bacteria (13%) (Supplementary Table 1). A decrease of 6% relative abundance of *Marinobacter* was observed to have occurred in the ambient CO_2_ microcosms (reaching 15% abundance) after 7 days of oil enrichment. However, an increase of 9% was observed in the elevated CO_2_ treated microcosms (reaching 24%), while *Methylobacterium* decreased by 1% to 3% in the elevated CO_2_ microcosms (Supplementary Table 1).

After just one day since the addition of crude oil to the ambient CO_2_ treatments, major increases in relative abundances were observed for members of unclassified Bacteria (by 12%) and unclassified *Halomonadaceae* (by 5% from undetectable levels), whereas minor increases occurred with *Methylobacterium* and unclassified *Rhodobacteraceae* (3% each), *Methylophaga*, *Corynebacterium* and unclassified *Alphaproteobacteria* (2% each), and *Sphingomonas*, *Stappia* (from undetectable levels) and *Halomonas* (1% each) (Supplementary Table 1). Within this same short time period since the addition of the oil to the elevated CO_2_ treatments, major increases in relative abundances were observed for *Marinobacter* (9%), *Prevotella* (7%), *Streptococcus* (6%) and *Veillonella* (5% from undetectable levels), with minor increases by *Gracilimonas* (4% from undetectable levels), *Stappia* (from undetectable levels), unclassified *Veillonellaceae* (from undetectable levels), *Sulfitobacter* and unclassified *Gammaproteobacteria* (3% each), and *Campylobacter* (from undetectable levels) and *Fusobacterium* (2% each) (Supplementary Table 1). At the end of these microcosm incubations that represented 14 days after the addition of the oil (t_2_), notable dynamics of specific taxa were observed for *Methylobacterium* which maintained a higher relative abundance in the ambient CO_2_ incubations without oil, whereas *Marinobacter* maintained a high relative abundance (15–29%) in both the ambient (without oil) and the elevated (with oil) CO_2_ treatments.

### Degradation of crude oil under ambient versus elevated CO_2_ conditions

**Figure 4:**
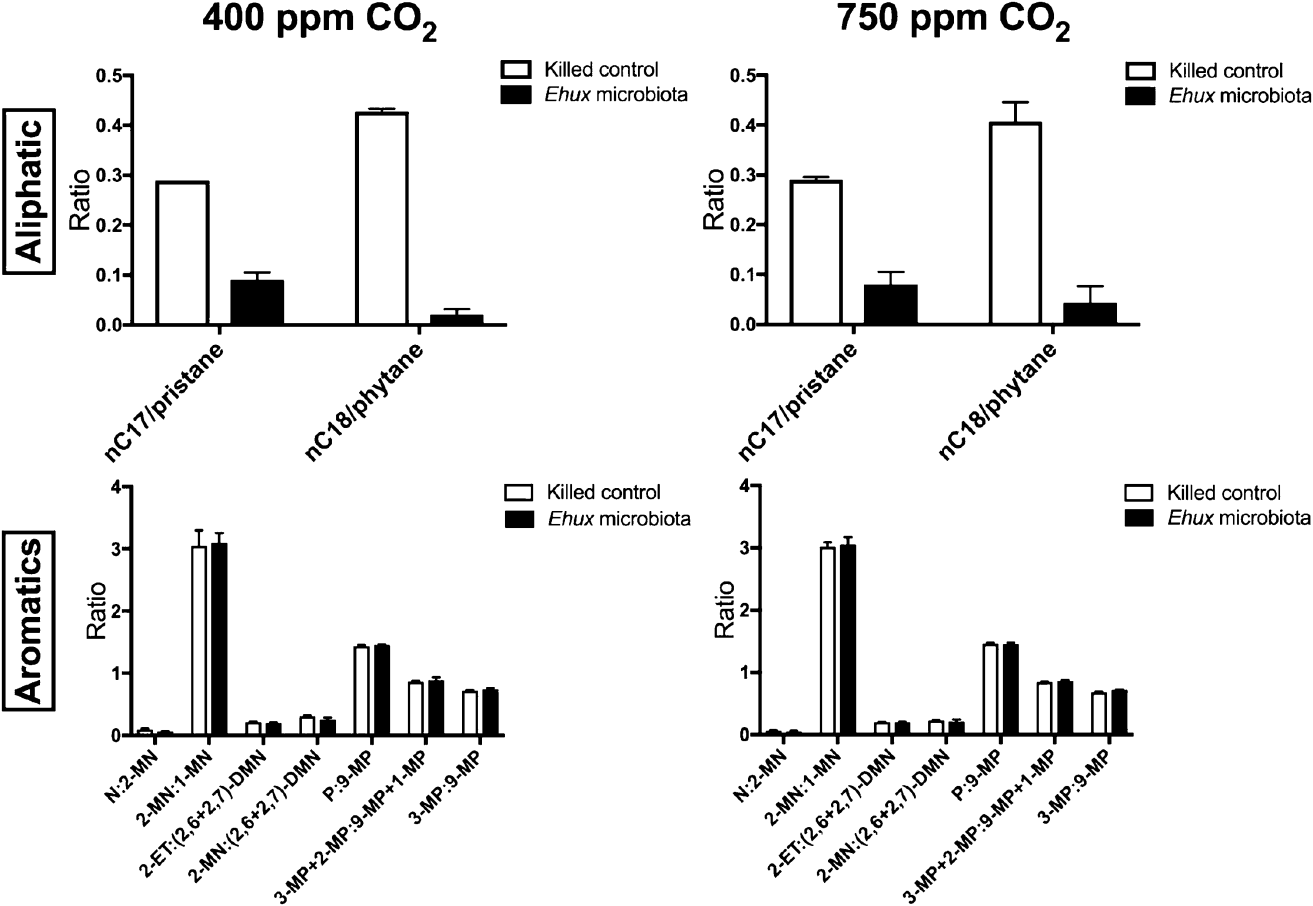
Differences in hydrocarbon ratios comparing live treatments of *E. huxleyi* with its associated bacterial community (solid bars; *Ehux* microbiota) to their respective acidified controls (open bars; killed controls) for parameters indicative of biodegradation: *n*C17/pristine, *n*C18/phytane, naphthalene/2-methylnaphthalene (N:2-MN), 2-methylnaphthalene/1-methylnaphthalene (2-MN;1-MN) 2-ethylnaphthalene/2,6 + 2,7-dimethylnapthalene (2-ET:(2,6 + 2,7)-DMN), phenanthrene/9-methylphenanthrene (P:9-MP), 3-methylphenanthrene + 2methylphenanthrene/9-methylphenanthrene + 1-methylphenanthrene (3-MP + 2-MP:9-MP + 1-MP), 3-methylphenanthrene/9-methylphenanthrene (3-MP:9-MP). Values are averages of triplicate incubations with standard error bars.

Of 35 *n*-alkanes analysed (*n*C8–*n*C43), thirteen (*n*C15–*n*C28) were biodegraded by the bacterial community associated with *E. huxleyi*, with significant differences detected for *n*C17/pristane and *n*C18/phytane between killed controls and inoculated samples (*p* < 0.0001) at the termination of the experiment (Fig. 4; Supplementary Fig. 3), thus indicating biodegradation of crude oil by the bacterial community associated with *E. huxleyi*. However, no significant differences (*p* < 0.05) of these aliphatic biomarkers was found in neither the ambient nor elevated CO_2_ treatments, suggesting that biodegradation of aliphatic hydrocarbons in crude oil is not affected by elevated CO_2_ conditions. Lower molecular weight *n*-alkanes, which includes *n*C8–*n*C10, were not detected in acid-killed controls and *E. huxleyi* cultures likely due to their evaporation over the course of the experiment.

Reductions of aromatic compounds, which include naphthalene/2-methylnaphthalene (N:2-MN), 2-methylnaphthalene/1-methylnaphthalene (2-MN;1-MN) 2-ethylnaphthalene/2,6 + 2,7-dimethylnapthalene (2-ET:(2,6 + 2,7)-DMN), phenanthrene/9-methylphenanthrene (P:9-MP), 3-methylphenanthrene + 2methylphenanthrene/9-methylphenanthrene + 1-methylphenanthrene (3-MP + 2-MP:9-MP + 1-MP), 3-methylphenanthrene/9-methylphenanthrene (3-MP:9-MP), were not detected after 15 days of exposure with *E. huxleyi* and its associated bacterial community (Fig. 4). Although the bacterial community associated with *E. huxleyi* had only reduced the low-molecular-weight fraction of *n*-alkanes such as pristane and phytane, but not aromatics within 15 days of crude oil enrichment, elevated CO_2_ conditions did not significantly affect the *n*-alkanes degradation of crude oil in inoculated samples.

## Discussion

This study shows, for the first time, the effects of crude oil on *E. huxleyi* and its associated bacterial community under future OA (elevated CO_2_) conditions. Initially, we show that OA enhanced the growth of *E. huxleyi*, as elevated CO_2_ resulted in higher Chl *a* concentrations (a proxy for growth of microalgae) and this is consistent with some previous studies^36,37^. However, crude oil induced a negative response, and together with elevated CO_2_, both conditions appeared to have a synergistic affect upon the growth of *E. huxleyi*. CO_2_ is a limiting factor in photosynthesis, therefore higher concentrations can be utilised to compensate for the metabolic stress of maintaining intracellular pH and especially as cells divide and replicate. Whilst the cells may grow in more abundance under elevated CO_2_, we posit that their coccoliths might be less calcified due to the relatively lower pH. If this were the case, it could explain their higher susceptibility to crude oil toxicity. *E. huxleyi* was reported to adopt the ‘Cheshire Cat’ escape strategy where the cells exist predominantly in a haploid stage that lack in mineralised scales, allowing evasion of viral predation in addition to physical and chemical stress^38–40^. Further work would be needed to ascertain whether calcification provides a physical defence against crude oil toxicity, or if the converse (i.e. a lack of mineralised scales) puts the organism at risk of being "killed" when challenged with crude oil. Hydrocarbon adsorption on calcite surfaces has been reported^41^. As such, it may be conjectured that thicker, healthier calcite scales would prevent, or might at least reduce the absorption of toxic hydrocarbon chemicals through and into the cell membrane and intracellular region of the cells where it would inevitably cause impairment of cell functioning and ultimately death. As discussed below, the associated bacterial community, and more specifically the oil-degrading community will play a protective role from crude oil toxicity.

Community analysis of the various treatments showed that bacterial species diversity associated with *E. huxleyi* was not significantly different neither under elevated compared to ambient CO_2_ conditions, nor with/without exposure to crude oil. However, certain shifts in the communities, as effected by certain taxa, were observed that delineated these treatments, as observed with the transient enrichment of *Marinobacter*, *Methylobacterium* and unclassified Bacteria in the ambient CO_2_ treatments after exposure to crude oil. *Marinobacter* is a genus known to utilize aliphatic and polycyclic aromatic hydrocarbons as a sole source of carbon and energy^42–44^. Whilst *Marinobacter* are commonly associated with the degradation of aliphatic and aromatic hydrocarbons in the ocean, they are in fact also capable of utilizing various non-hydrocarbon substrates, such as organic nitrogen-containing compounds (e.g. amino acids, carboxylic acids etc.)^45^. A decrease in their relative abundance (0–19%) after exposure to the oil was likely due to increased competition for bioavailable macro- and/or micronutrients. Although primary production increases production of organic carbon, photosynthesis utilises micronutrients such as iron that are co-limiting growth factors for heterotrophic bacteria^3,46,47^. Not unexpectedly, *Marinobacter* increased in relative abundance after oil enrichment and primarily under elevated CO_2_ conditions which has not been previously reported. We posit that under future OA conditions, these ubiquitous oil-degrading organisms may not be expected to be affected in their natural response to oil spillage.

Unlike *Marinobacter*, under elevated CO_2_ conditions *Methylophaga* and *Methylobacterium* became negatively, albeit marginally, impacted following exposure to crude oil, whereas these organisms thrived under the ambient CO_2_ treatments after exposure to the oil. Whilst some methylotrophs have been shown to become enriched by crude oil or its petrochemical refined products [42 and references therein], hitherto this has not been reported for members of the genus *Methylobacterium*. This genus belongs to a group of organisms known for methylotrophy, in that they rely exclusively on single carbon compounds, such as methanol, methylamine and dimethylsulfide, as a sole source of carbon and energy^48^. However, genes have been identified within *Methylobacterium* genomes that are associated with the degradation of hydrocarbons^49^, and the genome annotation for *Methylobacterium extorquens* PA1 in the KEGG (http://www.genome.jp/kegg-bin/show_pathway?mex01220) has been shown to possess anaerobic benzene degradation genes. Some methylotrophs, particularly of the genus *Methylophaga*, are also capable of utilising fructose^50^ or even hydrocarbons^51,52^ as sole growth substrates. Furthermore, the presence of hydrocarbon-degrading methylotrophs has been shown amongst communities of bacteria associated with eukaryotic phytoplankton^27,53^. This might be because eukaryotic phytoplankton can adsorb hydrocarbons from the seawater environment or because they synthesise alkenones and other hydrocarbon-like compounds [^54^ and references therein] which hydrocarbon-degrading methylotrophs could potentially feed on, although this hydrocarbon-mediated algal–bacterial symbiosis remains unproven. Whilst it remains to be substantiated, we posit that the enrichment of *Methylobacterium* in the presence of crude oil under ambient CO_2_ levels may be due to their potential to utilise hydrocarbons as a source of carbon and energy. An alternative explanation for their enrichment may be because these organisms were able to acquire their carbon and energy requirements from extracellular particulate and/or dissolved organic carbon (DOC) released by *E. huxleyi*; such exudates can offer a rich source of methylated sugars that can be utilised by methylotrophs^55,56^. It is also noteworthy to mention the release of organic matter exudates by *E. huxleyi* has been reported to be affected by changes in dissolved CO_2_ levels^57^. Whilst we did not analyse for exudate production in our experiments, others showed that elevated CO_2_ conditions significantly increased the release of transparent exopolymer particles (TEP) and particulate combined carbohydrates (pCCHO) by *E. huxleyi*^57^. Although the lability of this carbon source to be utilised by methylotrophs, like *Methylobacterium*, is unknown. At the very least, *Methylobacterium* was able to survive when challenged with the crude oil, but only under ambient CO_2_ conditions. As explained earlier, the coccolith scales of *E. huxleyi* would be in a weakened state, or less robust, to deal with oil exposure under elevated CO_2_ conditions, due to the detrimental effects caused by a reduced pH to the structure of their calcium carbonate armour, potentially permitting the intracellular entry of toxic hydrocarbons.

*Sphingomonas* are metabolically diverse and capable of utilising hydrocarbons as growth substrates^58^, but these organisms were not enriched after oil exposure under ambient CO_2_ conditions, and their relative abundance had noticeably decreased after oil exposure in the elevated CO_2_ treatment. This genus belongs to the class *Alphaproteobacteria*, and their decrease in relative abundance under elevated CO_2_ supports studies that found that OA conditions (from elevated CO_2_ levels) can negatively impact members belonging to this class^59,60^. Other genera comprising members with reported hydrocarbon-degrading abilities, such as *Halomonas* and *Alteromonas*, and which have been reported associated with *E. huxleyi*, such as *Marivita, Hoeflea, Balneola, Arenibacter, Marinoscillum* and *Thalassospira*^22^, were also detected but not affected by either CO_2_ treatment and nor exposure to the oil. We suspect that these organisms found associated with *E. huxleyi* are incapable of utilising hydrocarbons, which might explain why they were not enriched by the oil in either of the CO_2_ treatments. On the other hand, members of the genera *Prevotella*, *Streptococcus* and *Veillonella* which have been reported with hydrocarbon-degrading ability^61–64^, had increased in relative abundance following oil enrichment, but only under the elevated CO_2_ treatment, but then declined to < 1% abundance by the termination of these experiments. It is unknown to us at present what may have caused this post-bloom decline of these taxa and why these changes only occurred in the elevated CO_2_ treatment. It is possible their decline unfolded as a consequence of an essential nutrient becoming limited, accumulation of a toxic byproduct(s) in these microcosms, or other factor(s) that could be explored in future studies.

With atmospheric CO_2_ concentrations continuing to unabatedly rise and drive OA, our results show that the structuring of bacterial communities living in association with coccolithophores will be quite different in a future ocean, including potential extinctions of some oil-degrading taxa from these communities. Consequently, it is expected that this could have quite profound effects on the fate of spilled crude oil in the ocean, whether from natural seepage or anthropogenic inputs, considering that oil-degrading bacteria are crucial in the biodegradation and ultimate purging of petrochemical pollutants in the ocean. Assessing this by comparing *n*C17/pristane and *n*C18/phytane ratios of acid-killed incubations with the same from live incubations, we found that the biodegradation of the aliphatic fraction occurred under both ambient and elevated CO_2_ conditions. Despite shifts in microbial population dynamics due to elevated CO_2_ conditions, aliphatic hydrocarbon degradation was unaffected in both ambient and elevated CO_2_ conditions tested. We note that *n*C8, *n*C9, and *n*C10 were not detected in all treatments, likely due to their loss by volatilisation as expected for these low-molecular-weight hydrocarbons. On the other hand, the aromatic fraction in the crude oil, as measured by our analysis of naphthalene, methylnaphthalene, dimethylnaphthalene, ethylnaphthalene, C3-alkylnaphthalene, phenanthrene, methylphenanthrene, dimethylphenanthrene, ethylphenanthrene and C3-alkylphenanthrene, was not significantly degraded in neither the ambient or elevated CO_2_ conditions. It is possible that the oil-degrading microbial community associated with *E*. *huxleyi* was, for some reason, incapable of inducing the degradation of aromatic hydrocarbons. The metabolic capability to degrade aromatic hydrocarbons, however, would likely have been served by *Marinobacter*^65–68^, as members of this genus have been reported to utilise polycyclic aromatic hydrocarbons as sole growth substrates. Our experiments were run for 22 days, which was likely too short to begin to detect the initial stages in the biodegradation of the aromatic fraction because, as has been reported in many marine oil spill studies, the biodegradation of the aromatic fraction in crude oil does not often commence until the more labile aliphatics have become almost depleted (e.g. Head et al.^69^). Whilst we cannot conclude on what effects OA conditions might have on the biodegradation of aromatic hydrocarbons, based on our analysis of the aliphatic fraction, our results suggest that the response and biodegradation activities of oil-degrading communities associated with coccoliphores, such as *E. huxleyi*, would be largely unaltered when challenged with crude oil in future OA conditions.

## Conclusion

The study presents evidence that in the event of an oil spill under future OA conditions, *E. huxleyi* becomes highly vulnerable, whereas its associated bacterial community, with the exception of a few taxa, are resilient. *Marinobacter* was the dominant bacteria associated with *E. huxleyi* prior to oil-enrichment in both ambient and elevated CO_2_ conditions, and in elevated CO_2_/oil-enriched conditions up to 16 days after oil exposure. Only under ambient/oil-enriched conditions were *Marinobacter* overtaken by unclassified bacteria as the dominant taxa. Significant changes in population dynamics after oil enrichment between CO_2_ treatments where relative abundance of *Sphingomonas* and *Methylobacterium* from the class *Alphaproteobacteria* were reduced, did not significantly affect degradation of pristane and phytane from crude oil. Changes in bacterial population, though small, can be crucial to host survival. Recovery of an ecosystem from oil pollution under projected future ocean conditions depends not just on the potential for hydrocarbon degradation, but also on the microalgal host survival and primary production. Therefore, in a natural community of phytoplankton, where multiple species of microalgae and their microbiota co-exist and interact with each other, it is important to assess how primary producers and their bacterial community respond to crude oil enrichment under projected OA conditions since they are the foundation of an ecosystem.

## Methods

### Organism, maintenance and preparation of CO_2_-acclimatised inocula

A non-axenic *Emiliania huxleyi*, strain 920/8, was obtained from the Culture Collection of Algae and Protozoa (CCAP; Oban, Scotland). The strain was maintained in f/2 algal medium^70^ and in a temperature-controlled illuminated incubator as per the guidelines of the CCAP. The strain was originally isolated in 1992 from a Bergen mesocosm in Norway and in subsequent work it was found to harbour members of bacteria belonging to genera of obligate (e.g. *Alcanivorax*, *Marinobacter*) and generalist oil-degrading genera (e.g. *Arenibacter*, *Sulfitobacter*, *Thalassospira*)^22^.

*E. huxleyi* acclimatised to either ambient or elevated CO_2_ levels were prepared for use as inocula. Cultures were pre-conditioned at 14 °C with ambient light (50 μmol m^−2^ s^−1^) at a 16:8 h light:dark cycle. Bubbling of CO_2_ at ambient or elevated CO_2_ directly into the cultures was used to achieve soluble CO_2_ concentrations of 400 or 750 ppm, respectively. A 750 ppm concentration is the expected moderate projection for atmospheric CO_2_ by 2100, a value midway between SRES scenario A1B and A2^71^, and has been used in numerous algal-based ocean acidification studies. Ambient air was supplied from an air pump connected to outside air, while elevated CO_2_ was mixed using mass flow controllers to control the flow rate of ambient air or of compressed CO_2_. CO_2_ levels determined for elevated conditions were measured and monitored constantly using a COZIR ambient sensor (Gas Sensing Solutions Ltd., Glasgow, UK) calibrated with premixed 750 ppm CO_2_ (CalGaz Ltd., Newcastle, UK). CO_2_ readings were recorded per second and daily averages were calculated using R software. *E. huxleyi* was acclimatised for 100 generations under the two CO_2_ conditions (400 and 750 ppm) prior to use in experiments.

Supplementary Figure 4 shows the daily average CO_2_ concentrations during 20-day incubations supplemented with elevated atmospheric CO_2_, producing a CO2 concentration linear regression (standard curve) of 740 ppm. Two-way ANOVA analysis of pH in microcosms inoculated with or without *E. huxleyi* (Supplementary Fig. 5) shows significant differences between the treatments (*p* < 0.05), and between time and treatment (*p* < 0.005). A Tukey’s multiple comparison test showed that just prior to oil addition on day 5, pH levels were significantly different in ambient and elevated CO_2_ treated microcosms inoculated with *E. huxleyi* (*p* = 0.0107) and on day 20 (*p* = 0.0174; Two-Way ANOVA).

### Microcosm setup

Microcosms were set up using 500 ml Duran Youtility bottles with GL-45 4-inlet screw caps (Supplementary Fig. 6). Two of the 4-inlets were used for channelling air in and out of the bottles. Each microcosm was fitted with a 0.22 μm inline air filter attached to the inlet and outlet of the GL-45 bottle cap to prevent contamination. A glass Pasteur pipette connected to the inside of the GL-45 cap of each microcosm was used to aerate the system and also provide agitation by gentle bubbling. All materials that were in direct contact with the aqueous system inside the microcosms were made of glass in order to prevent the adsorption of hydrocarbons when crude oil was used.

Exponentially-growing cells of pre-acclimatised *E. huxleyi* were counted using a haemocytometer in order to standardise the inocula so that all microcosms received approximately the same cell concentration. A series of the sterilised Duran bottles, each containing 300 ml of sterile f/2 medium, were inoculated with standardised cell suspensions that were acclimatised to either ambient or elevated CO_2_ levels—final cell concentrations achieved across all microcosms were 8 × 10^3^ cells/ml. Microcosm treatments (each in triplicate) included (i) an oil-amended treatment inoculated with *E. huxleyi* for biological sampling, (ii) an oil-amended treatment inoculated with *E. huxleyi* for hydrocarbon analysis, (iii) a non-oil-amended treatment inoculated with *E. huxleyi* for biological sampling, and (iv) an acid-killed oil-amended control inoculated with *E. huxleyi* to account for loss of hydrocarbons due to abiotic factors, for which phosphoric acid was added to a final pH of ~ 1. The treatments were conducted under ambient and elevated CO_2_ conditions, and the complete setup totalled 24 individual microcosms. For the microcosm treatments designated for amendment with oil, the oil was added at day 5 to 1% (v/v) and Schiehallion crude oil (API 25°; sourced from BP) was used. All microcosms were incubated in a temperature-controlled incubator maintained at 14 °C.

The bottles were arranged equidistant from a central, vertical light source to ensure each microcosm received equal light intensity at a 16:8 h light:dark cycle. This arrangement avoids self-shadowing of microalgal cells, especially during their exponential growth which can lead to reduction of light intensity with increasing path length, as per Beer’s law, and shadowing from bottle caps and airline connections. AquaWhite flexi-LED (Aqualease Ltd., Blackburn, UK) strips were used to construct the light cylinders by wrapping them around a plastic tube for provision of a centralized vertical light source for each microcosm setup. Neutral density filters were used to reduce the light intensity to 58 µmol m^−2^ s^−1^.

Samples for chlorophyl *a* analysis and DNA extraction for monitoring the dynamics of *E. huxleyi* and the bacterial community by amplicon sequencing analysis were taken periodically from all microcosms, with the exception of the oil-amended incubations which were maintained unsampled until the end of the experiment, when they were sacrificed for hydrocarbon analysis (described below).

### Quantification of *E. huxleyi* dynamics by Chl *a* analysis

To assess the dynamics of *E. huxleyi* in response to crude oil exposure, changes in chlorophyll *a* (Chl *a*) were determined using a modified version of EPA 445^72^. For this, samples (2 ml) were centrifuged (13,000 xg; 10 min) and the cell pellets carefully transferred to a clean tube containing 1 ml of 90% acetone and stored in the dark at − 20 °C overnight. For samples derived from oil-amended microcosms, transferring the pellets to a clean tube helped eliminate the possibility of any carry-over from residual oil or solubilised hydrocarbons. After overnight storage, the samples were centrifuged and the supernatant fractions transferred into clean glass vials for fluorometric analysis at an excitation wavelength of 485 nm and emission of 685 nm using a Turner Trilogy Fluorometer (Turner Designs, CA, USA). Chl *a* concentrations were calculated from a standard curve constructed from serial dilutions of known Chl *a* concentrations (Turner Designs, CA, USA), as per the method of Welschmeyer^73^.

### DNA extraction, amplification and Illumina MiSeq sequencing

The diversity and response of the algal (*E. huxleyi*)-associated bacterial community to crude oil was assessed for each of the ambient and elevated CO_2_ treatments using Illumina MiSeq sequencing. This was performed at t_0_ (representing day 5 since the start of the incubations and just prior to oil enrichment), t_1_ (representing day 12 since the start of the incubations and 7 days after oil enrichment), and t_2_ (representing day 19 since the start of the incubations and 14 days after oil enrichment). Cell biomass from samples (5 ml) were taken at these three time points for extraction of whole genomic DNA. The samples were filtered using a vacuum filtration manifold (Millipore Sigma, UK) with 47 mm polycarbonate membrane filters (0.22 μm pore size) and the filters stored at − 20 °C. For extraction of DNA, the filters were crushed in liquid nitrogen to a fine powder and the nitrogen then allowed to evaporate off completely. DNA was extracted using the potassium ethyl xanthogenate method based on that by Tillett and Neilan^74^. Extracted DNA was confirmed using a Nanodrop 3300 fluorescence spectrometer (ThermoFisher Scientific, UK) and further confirmed by gel electrophoresis. PCR amplification of the V4 16S rRNA gene fragment was performed using Platinum Hot Start PCR Mastermix (ThermoFisher Scientific, UK), as described by the manufacturer’s protocol. Thermocycler conditions were 94 °C for 3 min, 35 cycles of 94 °C for 30 s, 50 °C for 60 s, and 70 °C for 90 s, and then a final extension step at 72 °C for 10 min.

Two to three replicates from each sample were selected and underwent amplicon sequence library preparation using Golay barcoded 515FB forward primers (5’-GTGYCAGCMGCCGCGGTAA-3’) and a complementary 806RB reverse primer (5’-GGACTACNVGGGTWTCTAAT-3’), according to the Earth Microbiome Project^75,76^. Libraries were pooled and sequenced at Edinburgh Genomics (Edinburgh, UK), using an Illumina Miseq sequencing platform employing the V2 (2 × 250) reagent kit. De-multiplexed and primer trimmed data files were returned for downstream processing, as described below.

Targeted sequencing of the modified V4 region using this pair of 515FB and 806RB improves detection of clade SAR11 in marine samples without affecting detection of taxa previously identified in region 515f/926r^75^.

### Sequencing data analysis

Libraries of bacterial community 16S rRNA gene sequences were constructed for OTU-based analysis using mothur version 1.39.5^77^. Contiguous sequences from paired-end sample reads were constructed, and homopolymers more than 8 bases long were removed. The sequences were then aligned against the SILVA database. Chimeras were removed and sequences that were left were classified and taxonomically referenced to the RDP database (version 16), and similarities below 80% were removed. Variation in species abundance and composition of bacterial communities associated with *E. huxleyi* in different treatments were analysed with non-metric multidimensional scaling (NMDS) and ANOSIM analysis. Shannon-Weiner diversity was calculated to determine the alpha diversity of treatments and a heat map was generated to visualise similarities of bacterial families in the different treatments. Dominant microbial taxa between treatments were visualised by heat tree plots using metacodeR^78^. Pairwise comparisons of the heat tree plots were generated by calculating taxon abundance with Wilcox rank-sum test, and Benjamini Hochberg (FDR) correction for multiple comparisons was used to determine significant differences of taxa between treatments.

### Hydrocarbon analysis

Microcosms used for hydrocarbon analysis were not sampled for biological analysis and remained untouched until the end of experiment. Each of these microcosms was sacrificed at day 20 for extraction of TPH using HPLC-grade dichloromethane (DCM) at an oil/water mix to DCM ratio of 2:1. The DCM fraction was removed and the oil/water mix re-extracted an additional 3 times. DCM fractions from each microcosm were pooled and further treated as previously described^27^ to obtain gravimetric data to calculate the original sample weight and the weight of oil remaining, and to prepare the TPH extracts for analysis of aliphatic and aromatic hydrocarbons. Briefly, GC-FID was used for the analysis of aliphatic hydrocarbons, with ratios of *n*C_17_/pristine and *n*C_18_/phytane used for differentiating biological degradation from weathering^79^. Similarly, aromatic ratios for biodegradation were compared for naphthalene/2-methylnaphthalene (N/2-MN), 2-methylnaphthalene/1-methylnaphthalene (2MN/1-MN), 2-ethylnaphthalene/2,6 + 2,7-dimethylnaphthalene (2-ET/(2,6 + 2,7)-DMN), 2-methylnaphthalene/2,6 + 2,7-dimethylnaphthalene, phenanthrene/9-methylphenanthrene (P/9-MP), 3 + 2-methylphenanthrene/9 + 1-methylphenanthrene (3-MP + 2-MP/9-MP + 1-MP), and 3-methylphenanthrene/9-methylphenanthrene (3-MP/9MP). These methods were followed as previously described^27^. One-way ANOVA was carried out on ratios to determine significant differences of hydrocarbon degradation between treatments.

### Statistical analysis

The two-way ANOVA test was used to analyse the differences in Chl *a* concentration of *E. huxleyi* as well as pH observed in *E. huxleyi* cultures across the CO_2_ treatments (i.e. ambient vs elevated CO_2_), oil-enriched treatments and treatment-time factors using PRISM 9 statistical software (Graphstats Technologies, Karnataka, India). Similarly, aliphatic and aromatic hydrocarbon ratios were also compared using Tukey’s multiple comparison test after two-way ANOVA analysis. To examine bacterial community abundance between treatments, Wilcox rank-sum test followed by Benjamini-Hochberg (FDR) correction for multiple testing was applied to median proportion of OTU reads.

## Supplementary Information

Supplementary Information.

## Data Availability

All data used and/or analyzed during the current study are presented in the article. For additional data requests, Tony Gutierrez (tony.gutierrez@hw.ac.uk) or Sebastian Hennige (s.hennige@ed.ac.uk) can be contacted.
