# Supplementary material for: Effect of ocean acidification on the growth, response and hydrocarbon degradation of coccolithophore-bacterial communities exposed to crude oil
Source: Sci Rep. 2023 Mar 27;13:5013. doi: 10.1038/s41598-023-31784-5 (PMC10042988; doi:10.1038/s41598-023-31784-5)
Supplement: Supplementary file 1 — Supplementary Information. [file 41598_2023_31784_MOESM1_ESM.docx]

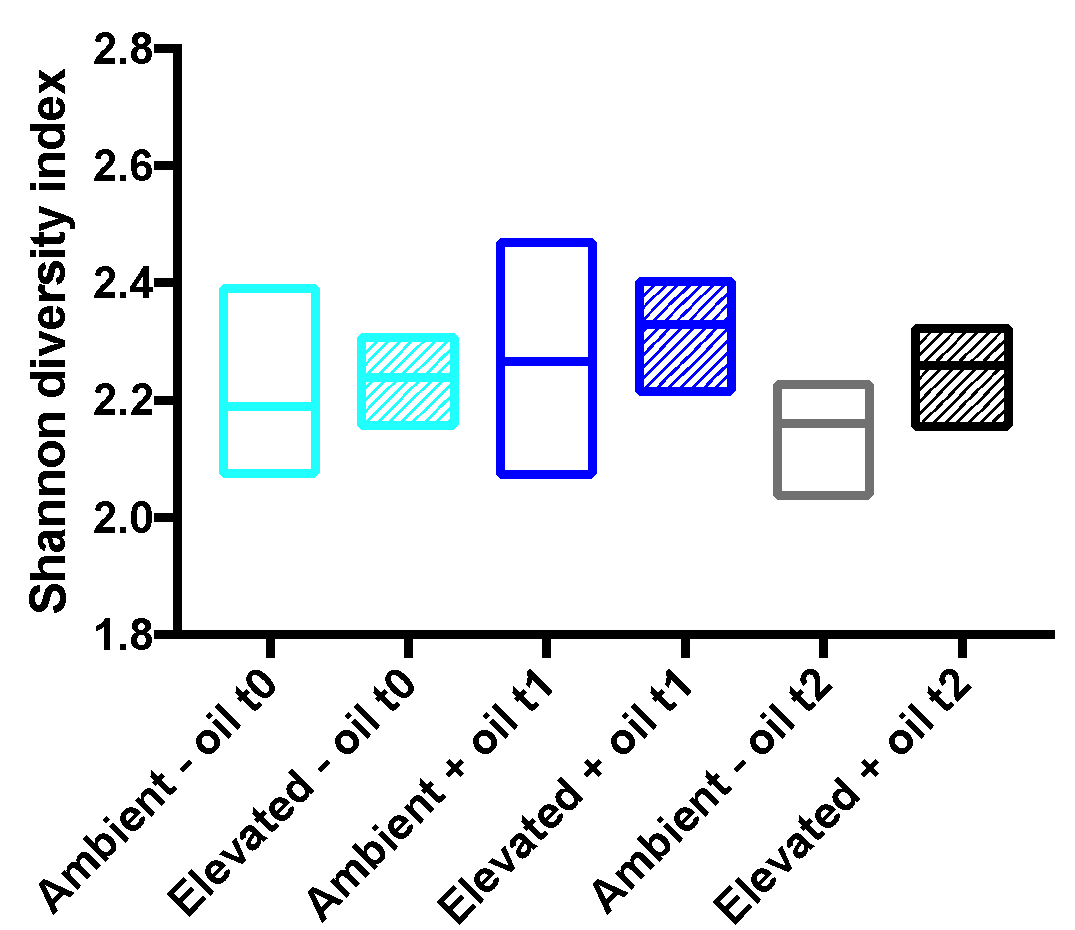


**Supplementary Figure 1.** Variance of OTU’s between treatments were analysed using Shannon diversity indices shown in floating bar plot. Line shows median value of Shannon diversity, with minimum and maximum values as borders of each bar plot. Clear bars indicate samples treated with ambient conditions at different timepoints, while cross-hatched bars represent microcosms treated with elevated CO_2_. Timepoints: t_0_ represents day 5 since the start of the incubations and just prior to oil enrichment, t_1_ represent day 12 since the start of the incubations or 7 days after oil enrichment, and t_2_ represents day 19 since the start of the incubations or 14 days after oil enrichment. - oil (no oil added); + oil (oil added).


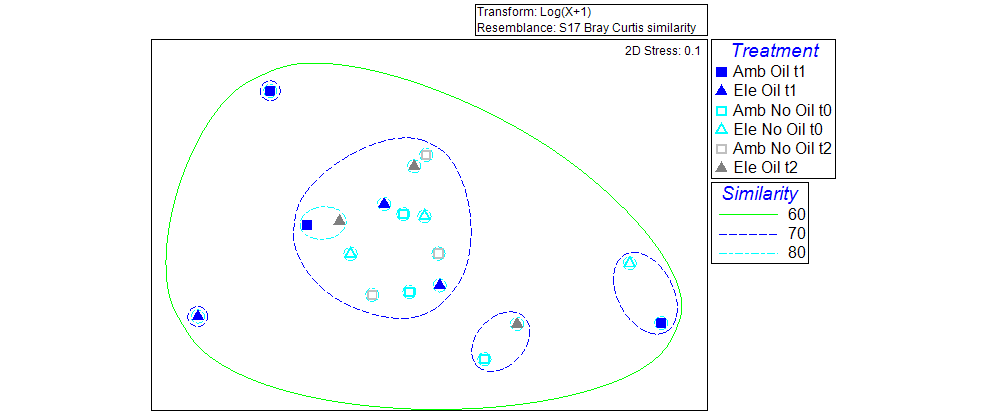


**Supplementary Figure 2.** nMDS plot based on Bray Curtis similarity index of the bacterial community associated with *E. huxleyi* in microcosms treated with/without oil and ambient or elevated CO_2_. Clusters based on log (X + 1) Bray-Curtis similarity index (%) are indicated by coloured ellipses. Timepoints: t_0_ represents day 5 since the start of the incubations and just prior to oil enrichment, t_1_ represent day 12 since the start of the incubations or 7 days after oil enrichment, and t_2_ represents day 19 since the start of the incubations or 14 days after oil enrichment. Amb (ambient CO_2_ conditions); Ele (elevated CO_2_ conditions).


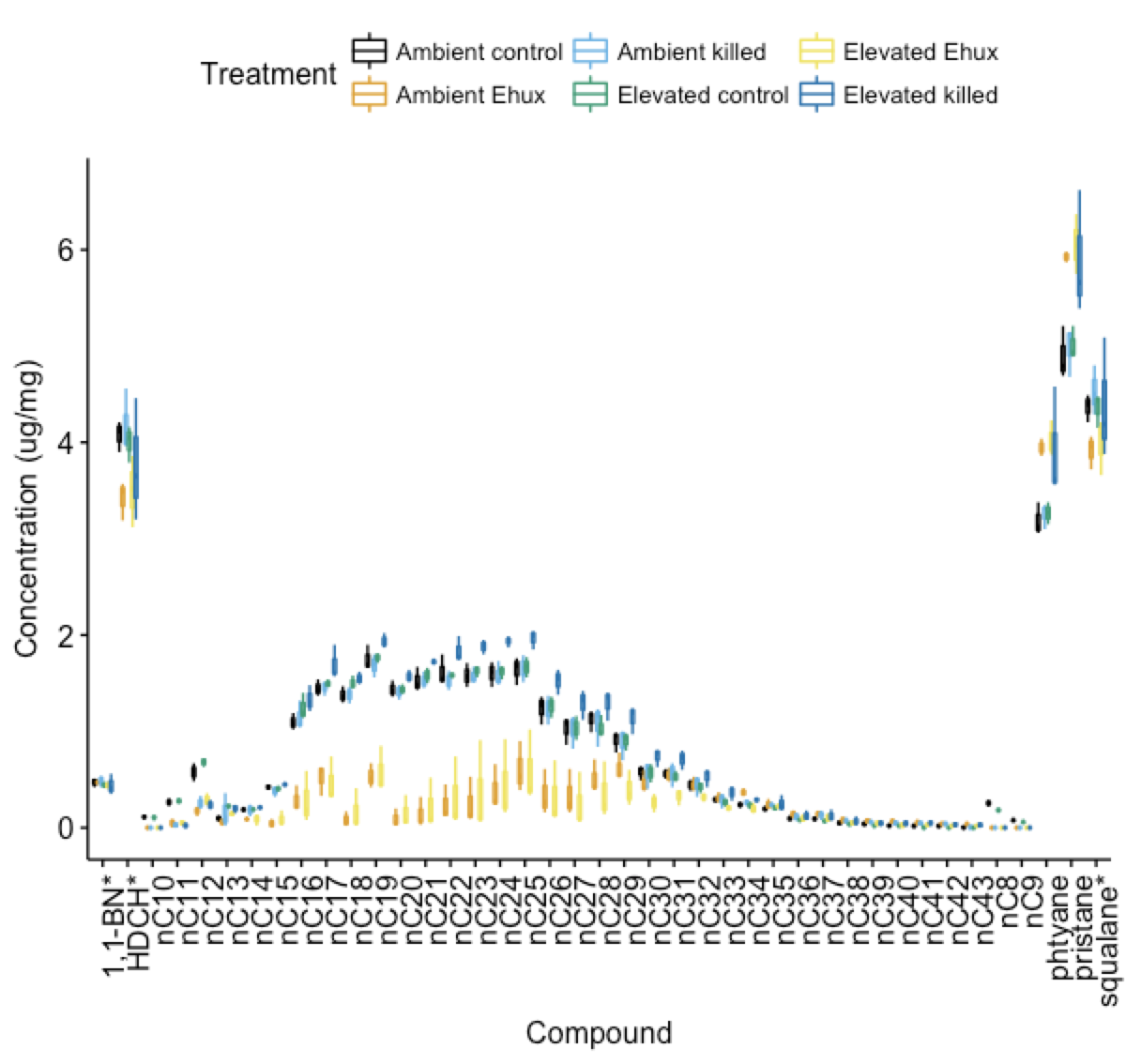


**Supplementary Figure 3**. Concentration of *n*-alkanes at the termination of the experiment (14 days after crude oil enrichment at day 5) in the following treatments that were amended with crude oil: Ambient control (400 ppm CO_2_); Ambient Ehux (400 ppm CO_2_ with *E. huxleyi*); Ambient killed (400 ppm CO_2_ with acid-killed *E. huxleyi*); Elevated control (750 ppm CO_2_); Elevated Ehux (750 ppm CO_2_ with *E. huxleyi*); Elevated killed (750 ppm CO_2_ with acid-killed *E. huxleyi*).


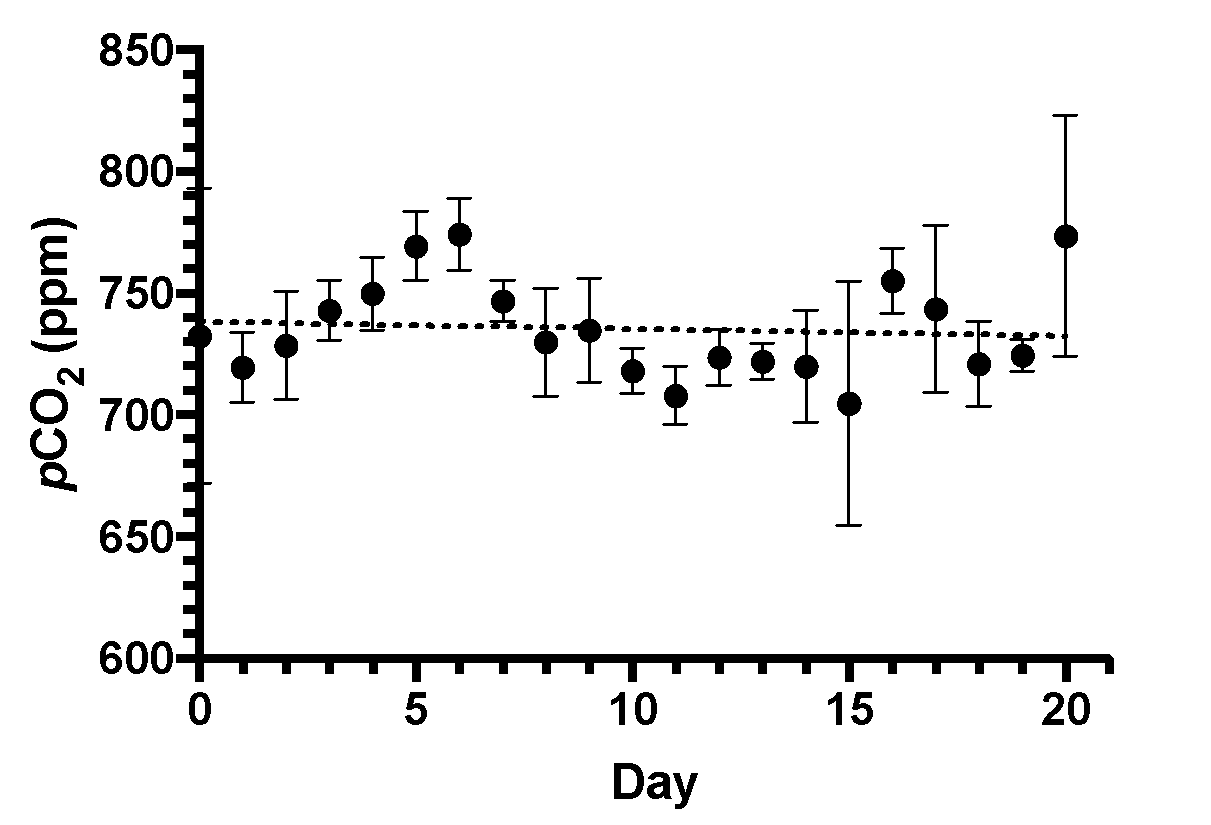


**Supplementary Figure 4.** Daily CO_2_ concentrations (ppm) measured in microcosms supplied with elevated *p*CO_2_. Linear regression (dotted line) shows the average CO_2_ concentrations. Values are the average of triplicate incubations ± standard error.


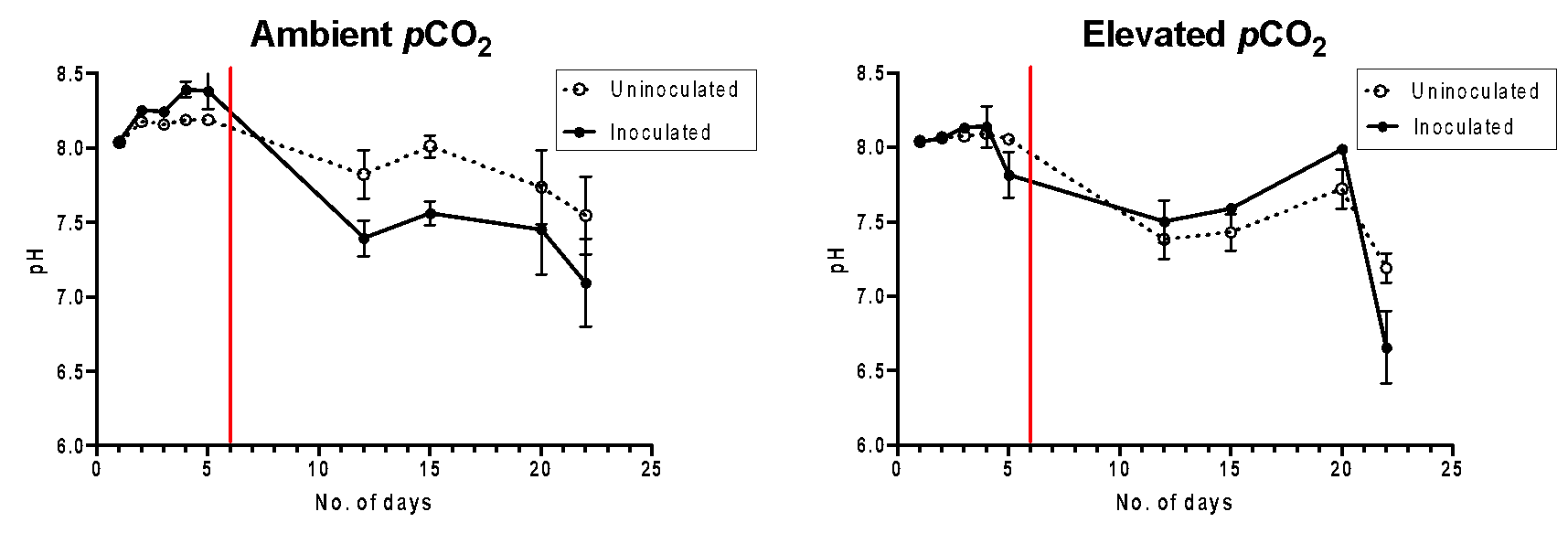


**Supplementary Figure 5.** pH levels of seawater in microcosms inoculated with (open circles) or without (closed circles) non-axenic *E. huxleyi* under ambient (400 ppm) and elevated (750 ppm) CO_2_ concentrations. Red line indicates the addition of crude oil to microcosms, with the sampling point at day 5 taken just prior to oil addition. Values are the average of triplicate incubations ± standard error.


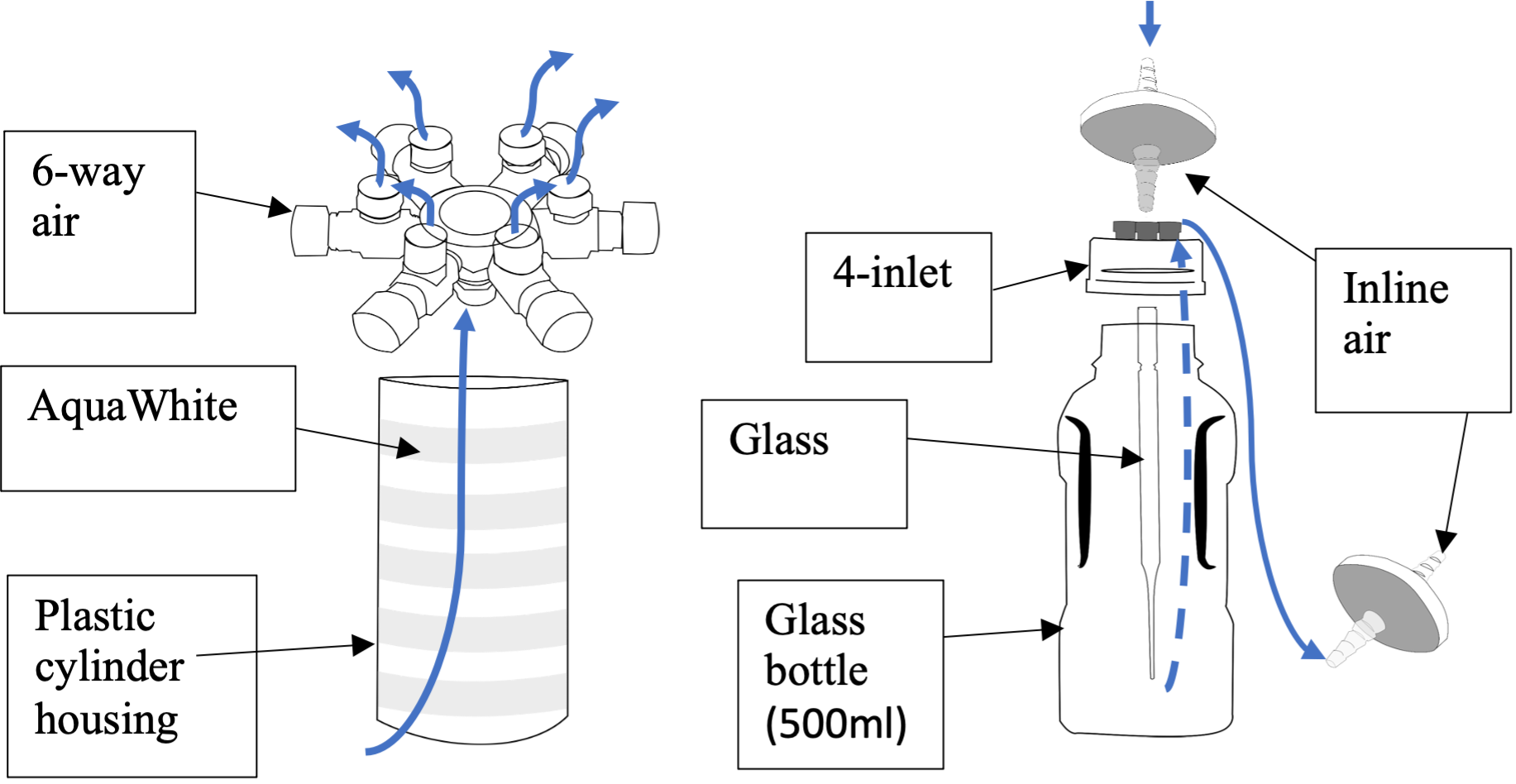


**Supplementary Figure 6**. Microcosm setup consisting of light and air supply component (left), and culturing vessel (right). Blue solid arrows indicate air flow connected to materials via airline tubing, while blue disconnected line shows air flow without tubing.

**Supplementary Table 1.** Percentage relative abundance (± standard error from triplicate incubations) of the bacterial community associated with *E. huxleyi* in microcosms incubated under ambient (400 ppm) or elevated (750 ppm) CO_2_ in the presence (+) or absence (-) of crude oil. Phylogenetic identification was assigned to the highest rank when possible. Timepoints: t_0_ represents day 5 since the start of the incubations and just prior to oil enrichment, t_1_ represent day 12 since the start of the incubations or 7 days after oil enrichment, and t_2_ represents day 19 since the start of the incubations or 14 days after oil enrichment.

| **Timepoint** | **T0** | | **T1** | | **T2** | |
| --- | --- | --- | --- | --- | --- | --- |
| **CO_2_** | **400 ppm** | **750 ppm** | **400 ppm** | **750 ppm** | **400 ppm** | **750 ppm** |
| **Oil enrichment** | **-** | **-** | **+** | **+** | **-** | **+** |
| Unclassified Bacteria | 6 ± 3 | 13± 8 | 18 ± 9 | 9 ± 6 | 16 ± 10 | 8 ± 2 |
| **Actinobacteria (Phyla)** | | | | | | |
| *Corynebacterium* | 1 ± 1 | 2 ± 1 | 4 ± 2 | 2 ± 1 | 2 ± 2 | 4 ± 1 |
| **Bacteroidetes (Phyla)** | | | | | | |
| *Prevotella* | 2 ± 0 | 1 ± 1 | 1 ± 1 | 8 ± 7 | 0 ± 0 | 0 ± 0 |
| *Balneola* | 4 ± 1 | 5 ± 1 | 3 ± 1 | 3 ± 2 | 5 ± 2 | 3 ± 0 |
| *Gracilimonas* | 2 ± 0 | 0 ± 0 | 0 ± 0 | 4 ± 2 | 1 ± 1 | 0 ± 0 |
| **Firmicutes (Phyla)** | | | | | | |
| *Streptococcus* | 3 ± 1 | 1 ± 1 | 0 ± 0 | 7 ± 4 | 1 ± 1 | 1 ± 0 |
| *Veillonella* | 1 ± 1 | 0 ± 0 | 0 ± 0 | 5 ± 3 | 2 ± 0 | 1 ± 1 |
| Unclassified *Veillonellaceae* | 0 ± 0 | 0 ± 0 | 0 ± 0 | 3 ± 2 | 0 ± 0 | 0 ± 0 |
| **Fusobacteria (Phyla)** | | | | | | |
| *Fusobacterium* | 0 ± 0 | 1 ± 1 | 0 ± 0 | 3 ± 2 | 0 ± 0 | 1 ± 1 |
| **Proteobacteria (Phyla)** | | | | | | |
| **Alphaproteobacteria (Class)** | | | | | | |
| Unclassified *Alphaproteobacteria* | 6 ± 2 | 4 ± 1 | 8 ± 5 | 3 ± 1 | 4 ± 1 | 5 ± 3 |
| *Maricaulis* | 4 ± 1 | 4 ± 1 | 2 ± 1 | 4 ± 2 | 4 ± 2 | 2 ± 1 |
| *Methylobacterium* | 8 ± 2 | 11 ± 4 | 11 ± 4 | 6 ± 3 | 10 ± 3 | 6 ± 0 |
| *Celeribacter* | 2 ± 0 | 2 ± 1 | 2 ± 1 | 1 ± 0 | 3 ± 1 | 2 ± 0 |
| Unclassified *Rhodobacteraceae* | 4 ± 1 | 5 ± 4 | 7 ± 1 | 3 ± 1 | 5± 4 | 4 ± 3 |
| *Stappia* | 0 ± 0 | 0 ± 0 | 1 ± 0 | 3 ± 2 | 1 ± 1 | 1 ± 1 |
|  |  |  |  |  |  |  |

| **Timepoint** | **T0** | | **T1** | | **T2** | |
| --- | --- | --- | --- | --- | --- | --- |
| **CO_2_** | **400 ppm** | **750 ppm** | **400 ppm** | **750 ppm** | **400 ppm** | **750 ppm** |
| **Oil enrichment** | **-** | **-** | **+** | **+** | **-** | **+** |
| **No. of OTU’s** | **89 ± 21** | **59 ± 25** | **45 ± 14** | **95 ± 32** | **52 ± 39** | **85±20** |
| *Sulfitobacter* | 3 ± 1 | 1 ± 1 | 2 ± 1 | 4 ± 2 | 4 ± 1 | 3 ± 1 |
| *Sphingomonas* | 5 ± 1 | 8 ± 2 | 6 ± 2 | 4 ± 1 | 6 ± 2 | 3 ± 1 |
| **Gammaproteobacteria (Class)** | | | | | | |
| Unclassified *Alteromonadaceae* | 3 ± 3 | 4 ± 3 | 2 ± 2 | 2 ± 2 | <1 ± <1 | 3 ± 3 |
| *Alteromonas* | 2 ± 0 | 3 ± 2 | 2 ± 1 | 1 ± <1 | 2 ± <1 | 3 ± 1 |
| *Marinobacter* | 29 ± 1 | 15 ± 4 | 15 ± 3 | 24 ± 8 | 20 ± 4 | 22 ± 5 |
| Unclassified *Gammaproteobacteria* | 11 ± 5 | 4 ± 1 | 5 ± 2 | 7 ± 3 | 8 ± 5 | 8 ± 4 |
| Unclassified *Halomonadaceae* | 0 ± 0 | 6 ± 6 | 5 ± 5 | 0 ± 0 | 0 ± 0 | 5 ± 5 |
| *Halomonas* | 2 ± <1 | 5 ± 2 | 3 ± 1 | 1 ± <1 | 2 ± 1 | 3 ± 1 |
| *Methylophaga* | 2 ± <1 | 4 ± 1 | 4 ± 2 | 2 ± 1 | 4 ± 1 | 3 ± 1 |
| **Epsilonproteobacteria (Class)** | | | | | | |
| *Campylobacter* | 0 ± 0 | 0 ± 0 | <1 ± <1 | 2 ± 2 | 1 ± 1 | 0 ± 0 |
